# Relationship between Inherent Cooking Rate and Warner-Bratzler Shear Force of Pork Chops Cooked to Two Degrees of Doneness

**DOI:** 10.3390/foods11010131

**Published:** 2022-01-05

**Authors:** Taylor N. Nethery, Dustin D. Boler, Bailey N. Harsh, Anna C. Dilger

**Affiliations:** 1 Department of Animal Sciences, University of Illinois, 1503 S. Maryland Drive, Urbana-Champaign, IL 61801, USA; taylor_nethery@barry-callebaut.com (T.N.N.); dboler2@illinois.edu (D.D.B.); bharsh2@illinois.edu (B.N.H.); 2 Topigs Norsvin USA, 12750 Nicollet Ave. S., Suite 300, Burnsville, MN 55337, USA

**Keywords:** cooking rate, degree of doneness, pork, tenderness, Warner-Bratzler shear force

## Abstract

The objective was to test inherent cooking rate differences on tenderness values of boneless pork chops when exogenous factors known to influence cooking rate were controlled. Temperature and elapsed time were monitored during cooking for all chops. Cooking rate was calculated as the change in °C per minute of cooking time. Warner-Bratzler shear force (WBSF) was measured on chops cooked to either 63 °C or 71 °C. Slopes of regression lines and coefficients of determination between cooking rate and tenderness values for both degrees of doneness (DoD) were calculated. Shear force values decreased as cooking rate increased regardless of DoD (*p* ≤ 0.05), however changes in tenderness due to increased cooking rate were limited (β1 = −0.201 for 63 °C; β1 = −0.217 for 71 °C). Cooking rate only explained 3.2% and 5.4% of variability in WBSF of chops cooked to 63 °C and 71 °C, respectively. Cooking loss explained the most variability in WBSF regardless of DoD (partial R^2^ = 0.09–0.12). When all factors were considered, a stepwise regression model explained 20% of WBSF variability of chops cooked to 63 °C and was moderately predictive of WBSF (model R^2^ = 0.34) for chops cooked to 71 °C. Overall, cooking rate had minimal effect on pork chop tenderness.

## 1. Introduction

Lack of consistency in tenderness values of boneless pork chops detrimentally affects consumers’ confidence in pork [1] and could be blamed for the lack of enthusiasm by US consumers for pork chops as a premium item. Several ante- and perimortem factors are known to influence tenderness. Factors such as pig diet [2] carcasses weight [3,4], and carcass chilling rate [5] all impact tenderness of boneless pork chops. Unfortunately, even when diet, carcass weight, chilling rate, and sire line are controlled, most variability in tenderness remains unexplained [6]. Even so, little attention has been paid to postmortem factors that may influence tenderness but occur outside of the control of meat producers, such as the rate of cooking of a product.

Relatively little is known about the relationship between cooking rate and tenderness or cooking loss in boneless pork chops. However, some information can be extrapolated from studies comparing cooking methods because different cooking methods resulted in different cooking times needed to reach a targeted internal temperature. Since cooking rate, as defined in this work, is the increase in temperature of the product per unit time, longer cooking times to reach an equal cooked temperature would result in a slower rate of cooking. Some studies have suggested that faster cooking methods resulted in more cooking loss and yielded tougher cooked products. Faster cooking methods, such as clam-shell and char-broil grills, resulted in beef steaks that were less tender and had greater cooking losses compared with slower methods, such as air-impingement ovens and forced-air convection ovens [7]. Similarly, forced convection plus steam cooking of pork loins resulted in faster cooking rates with more cooking loss compared with forced convection alone or natural convection [8]. However, all three methods resulted in similar tenderness values [8]. Ohmic cooking resulted in faster cooking rates than pan-frying pork chops though pan-fried chops were more tender. No differences in cooking loss were reported [9]. In contrast, others have determined that faster cooking methods yielded more tender products. Steam-cooking resulted in faster cooking rates, less cooking loss and more tender pork compared to griddle cooking [10]. The drawback of extrapolating cooking rate information from studies that use different cooking methods is that methods also vary in the temperature of the heat source, distance from the heat source, air movement, and contact with the heat source. These factors may affect tenderness independently of inherent differences in cooking rate. Therefore, while using different cooking methods suggests that cooking rate may alter tenderness in pork, these differences in rates are confounded with differences in cooking method. Instead, to isolate the effect of differences in cooking rate on pork chop tenderness, it would be critical to control all possible factors other than cooking rate and use a single cooking method.

Given the variability in composition of pork loin chops, the rate of cooking, even when using a single cooking method, could be expected to vary from chop to chop. Fresh pork loin chops are approximately 70–75% water, 18–20% protein, 2–3% ash, with the balance being lipid. As lipid increases in the form of marbling, water is reduced. Proximate composition, especially the content of water and lipid, can influence thermal conductivity. For example, the thermal conductivity of various dairy products was linearly related to water content and inversely dependent on fat content [11]. This suggests that products with more water and less lipid will heat more quickly than products with more lipid. Lipid content is variable in fresh pork loin chops. Visual marbling scores, a proxy for lipid content, of pork chops in the U.S retail market range from 1 to 6% [12]. Further, the lipid content of cooked pork chops can differ by as much as 6 percentage units, ranging from 1% to 7% in a recent study [13]. Given this variability, thermal conductivity, and therefore, inherent cooking rate, would be expected to differ among pork loin chops. While other factors, such as chop thickness and cooking surface temperature, also influence cooking rate, even when these are controlled, cooking rate may vary due to other factors inherent to the pork chops themselves. The hypothesis for this experiment was that chops with less marbling would have greater moisture and greater thermal conductivity. This increased thermal conductivity would result in faster cook rates, greater cooking losses, and a reduction in tenderness.

The objectives were to test the effects of inherent cooking rate differences on tenderness values of boneless pork chops, determine the relationships between inherent cooking rate differences and cook loss on instrumental tenderness values of boneless pork chops cooked to either 63 °C or 71 °C, and to determine if inherent differences in cooking rate improved the predictive ability of loin quality traits for tenderness. These two temperatures represented the recommended endpoint temperatures for whole-muscle pork cuts before (71 °C) and after (63 °C) the change in US recommendations [14].

## 2. Materials and Methods

Pork loins were purchased from a federally inspected processing facility; therefore, Institutional Animal Care and Use Committee approval was not necessary. Loins from three separate groups (day of slaughter) of barrows and gilts representing four sire lines were used in this study (503 total boneless loins). All four sire lines represented common breeding of commercially available pigs in the United States. Pigs from which loins originated were raised in commercial barns and fed diets commonly used in U.S. swine production systems under conditions typical of U.S. commercial swine production. Pigs were slaughtered when ending live weights ranged between 131 kg and 143 kg in 3 groups of about 160 pigs each over the course of 6 weeks.

### 2.1. Federally Inspected Abattoir Slaughter and Loin Selection

Pigs were transported to a single USDA (United States Department of Agriculture) federally inspected commercial abattoir and were held in lairage for a minimum of 3 h. Pigs were slaughtered under the supervision of the Food Safety and Inspection Service of the USDA. Pigs were immobilized using carbon dioxide and then terminated via exsanguination. Carcasses were blast chilled for approximately 90 min. Carcasses were allowed to equilibrate for a minimum of 22 h, prior to fabrication. Loins were fabricated into boneless Canadian back loins (NAMP #414) [15]. Loins were vacuum-packaged and boxed for transport to the University of Illinois Meat Science Laboratory. Loins were aged in refrigerated conditions (4 °C) until 14 d postmortem.

### 2.2. Aged Postmortem Loin Quality Evaluation

At 14 d postmortem, vacuum-packaged loins were weighed. Loins were then removed from packaging, allowed to drip for approximately 15 min., and weighed again. An average bag weight was collected by weighing 20 empty bags after being cleaned of any remaining purge to determine the average package weight. Purge loss (%) was calculated using the following equation: ((bagged loin wt.-wt.of bag)-loin wt.)/(loin wt.) × 100

Quality traits were evaluated by trained University of Illinois personnel following the procedure outlined by [16] on the ventral surface of the loin, posterior to the 10th rib. Instrumental lightness (L*), redness, (a*), and yellowness (b*) [17] were measured with a Minolta CR-400 Chroma meter (Minolta Camera Co., Ltd., Osaka, Japan) using a D65 illuminant, 2° observer angle, an 8 mm closed aperture, and calibrated using a white tile. Ultimate pH was measured by penetrating the ventral surface of the loin muscle in the approximate location of the 10th rib with a Hanna pH meter (model HI98163, Hanna Instruments Woonsocket, RI, USA). Visual color and marbling scores [18], and subjective firmness scores [19] were determined by a single technician. Chops were objectively evaluated for moisture percentage using the oven-drying method 950.46 described by AOCS International [20]. Extractable lipid was determined by first trimming pork chops of all subcutaneous fat and mincing into a homogenous consistency. Then chops were washed multiple times with warm chloroform and methanol [21]. Lipid percentage was calculated as the difference in the weight of a 10 g sample before and after washing in chloroform: methanol and redrying.

After quality evaluations, chops were collected from each loin posterior to the 10th rib for further analysis. Loins were sliced into 2.54 cm thick chops using a Treif Puma 700F slicer (Treif USA, Shelton, CT, USA). All chops were individually vacuum packaged and stored frozen at −20 °C until evaluation. The first chop posterior to the 10th rib from each loin was designated for another study. For groups 1 and 2, the second and third chops posterior to the 10th rib from each loin were collected and allotted to endpoint temperature groups of 63 °C and 71 °C, respectively. After cooking, these chops were used for determination of Warner–Bratzler shear force (WBSF) and cooking loss. From group 3, the second chop posterior to the 10th rib per loin was collected for WBSF and cooking loss after cooking to 63 °C. No chops from the third group were cooked to 71 °C due to experimental constraints. Chops were individually vacuum packaged and stored at −20 °C until analysis.

### 2.3. Cooking Procedures, Temperature, Cooking Loss and Warner-Bratzler Shear Force

Loins, within group (slaughter group), were allotted to analysis days so that chops from the same loins were cooked to both endpoint temperatures on the same day. Approximately 50 chops were cooked on each analysis day with a total of 8 d of cooking for group 1 and 8 d for group 2. For group 3, approximately 50 chops were cooked on each of 4 analysis days.

All chops were cooked on Farberware Open Hearth grills (model 455N, Walter Kidde, Bronx, NY, USA). These grills consist of an electric heating element that sits inside a deep rectangular metal base. Stainless steel grates (food contact surface) fit in notches in the top of the metal base and the grate sits above the heating element. There was a 4.45 cm space between the heating elements and the grates on the grills. These grills do not have adjustable temperature settings. Grills were pre-heated for a minimum of 10 min. prior to cooking each day. When heated, the heating elements ranged in temperature from 271 to 369 °C, and grates ranged in temperature from 163 to 197 °C. A total of 4 grills were used on each cooking day with 2 chops cooked on each grill at a time.

Chops were removed from the freezer 18 h prior to analysis and allowed to thaw thoroughly at approximately 4 °C prior to cooking. Chops were individually weighed prior to cooking. For groups 1 and 2, internal temperature was monitored using beaded wire thermocouples (type K, range: −200 °C to 1250 °C, standard error: ±2.2 °C, Omega Engineering, Stamford, CT, USA) placed in the approximate geometric center of chops and connected to a handheld digital scanning thermometer (Omega HH378 Data Logger Thermometer, Omega Engineering, Norwalk, CT, USA). For group 3, internal temperature was monitored using copper-constantan thermocouples (type T, range: 0 °C to 200 °C, standard error: ±1.0 °C, Omega Engineering, Stamford, CT, USA) placed in the approximate geometric center of chops and connected to a Digi-sense digital scanning thermometer (model 92000–00, Barnat Co., Barrington, IL, USA).

Initial temperature was recorded prior to placing chops on the grill after thermocouples were inserted and allowed to equilibrate. Chops grilled to 63 °C were cooked on one side to an internal temperature of 31.5 °C, flipped, and then cooked until they reached an internal temperature of 63 °C. Chops grilled to 71 °C were cooked on one side to an internal temperature of 36 °C, flipped, and then cooked until they reached an internal temperature of 71 °C. Temperature was recorded when chops were removed from the grill (off-grill temperature). Chops were allowed to rest with thermocouples still inserted and the peak resting temperature of chops were recorded (final temperature). For each individual chop, the total cooking time (time elapsed from when chops were placed on the grills until they were removed from grills) was recorded in s. Cook rate was calculated by taking the change in temperature (°C) from initial to off-grill temperature divided by elapsed time from when chops were placed on the grill to when they were removed from the grill and was expressed as the change in degrees Celsius per minute of cooking time (°C/min).

Chops were allowed to cool to approximately 25 °C and weighed again to determine percent cooking loss. Percent cooking loss was calculated using the following equation: (initial wt.-cooked wt.)/(initial wt.) × 100. Four 1.25 cm diameter cores were removed parallel to the orientation of the muscle fibers and sheared using a Texture Analyzer TA.HD Plus (Texture Technologies Corp., Scarsdale, NY, USA/Stable Microsystems, Godalming, UK) with a blade speed of 3.33 mm/s and a load cell capacity of 100 kg. The shear force value for the four cores were averaged and reported as WBSF.

### 2.4. Statistical Analyses

Summary statistics, including mean, sample variance, and CV, were calculated using the MEANS procedure of SAS (v.9.4, SAS Institute Inc., Cary, NC, USA). Pearson correlation coefficients between loin quality traits, initial chop weight, initial chop temperature, and cook rate were computed using the CORR procedure. Correlations were considered weak at r ≤ |0.35|, correlations were considered moderate at |0.36| ≥ r ≤ |0.67|, and strong correlations were those r ≥|0.68| [22]. Predictive ability of cook rate and cook loss on WBSF was determined using the REG procedure. An initial evaluation of the effect of slaughter group indicated differences in predictive ability of cook rate on tenderness. Therefore, the final regression model included an independent variable (either cooking rate or cooking loss) and slaughter group as a categorical variable. This resulted in estimates of slope for the independent variable and each set, as appropriate. Coefficients of determination (R^2^) and the slopes of each regression equation (β1) were considered significantly different from 0 at *p* ≤ 0.05.

A linear stepwise regression equation was developed using independent candidate variables to predict WBSF. Multicollinearity among 19 independent variables was assessed using a variance inflation factor (VIF) statistic and 5 variables (initial and final chop weight, hue angle, chroma, and cooking time) exceeded VIF values of 10. All other variables had VIF less than 7 and therefore, remained as candidate variables for selection in the model. Those variables included sire line, purge loss, ultimate pH, NPPC visual color, NPPC visual marbling, subjective firmness, L*, a*, b*, cooking loss, cooking rate, and chop temperature before cooking (initial), immediately after cooking (off-grill), and peak at resting (final). Influence of individual observations on the estimated dependent variable was determined using the difference of fit (DFITTS) statistic. Observations were considered to have excessive influence on the estimation of the regression parameters when DFITTS ≥ 2[(*x/n*) × 0.5] where x was the number of parameters considered and n was the total number of observations. No observations met this exclusion criteria. Using a stepwise selection method, independent variables were required to have a significant F statistic at the SLENTRY and SLSTAY level = 0.15 to be included and remain in the final model. Separate models for WBSF at 63 °C and 71 °C were generated.

## 3. Results and Discussion

Population summary statistics including mean, minimum observation, maximum observation, and CV were presented in Table 1. Ventral loin ultimate pH ranged from 5.4 to 6.0, ventral loin visual color scores ranged from 2.5 to 5.0, and ventral loin visual marbling scores ranged from 1.0 to 5.0. Ventral loin ultimate pH, visual color and marbling scores fell within similar ranges to those observed in the U.S. retain case [12] other large scale commercial pig studies [4,23]. A benchmark study by the National Pork Board reported that the average retail pork chop has an NPPC color of 3.11 and NPPC marbling score of 2.53 [12]. Therefore, while average pork loin color was slightly darker than average US retail pork, the ranges of quality measures in this study were representative of US pork observed in the retail situation. Thus, these loins were a good representation of commercially available pork.

Cooked chop traits are also presented in Table 1. Initial chop temperatures averaged 4.73 °C with a range of 0.3 °C to 11.8 °C for chops cooked to 63 °C and averaged 4.60 °C with a range of 0.8 °C to 14.7 °C in chops cooked to 71 °C. This supports the hypothesis that fresh pork chops differ in thermal conductivity. All chops (regardless of intended cooking temperature) used in this experiment were cut to the same thickness, allowed to temper at the same temperature and for the same duration. Still, variation in initial temperatures ranged over 14.4 °C (Table 1). Although there was variation in initial temperature of pork chops, the population average temperature reached targeted temperatures for refrigerated pork. Further, initial temperature was not correlated (*p* ≥ 0.44) with cooking rate regardless of targeted end-point cooking temperature (Table 2). For chops cooked to 63 °C, the average temperature of chops removed from grills (off-grill temperature) was 63.08 °C with a range of 63.0 °C to 67.2 °C. For chops cooked to 71 °C, the average temperature of chops removed from grills (off-grill temperature) was 71.04 °C with a range of 71.0 °C to 72.5 °C. Cooking times ranged from 13.54 min to 30.32 min for chops cooked to 63 °C, a difference of over 17 min, and 16.20 to 45.47 min for chops cooked to 71 °C, a difference of over 28 min.

**Figure 1:**
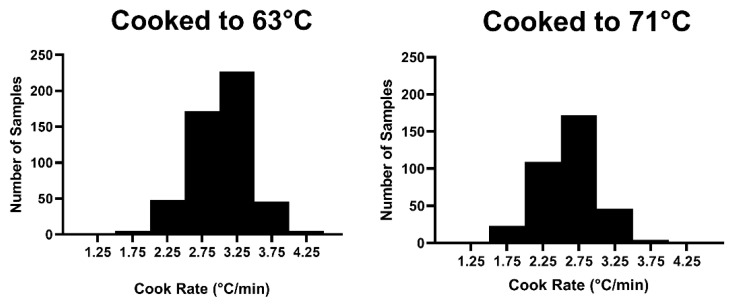
Histograms depicting the distribution of cooking rates for the population of chops cooked to (**Left**) 63 °C and (**Right**) 71 °C.

Clearly, based on the differences in elapsed cooking time, cooking rate varied for chops cooked to 63° and 71 °C. At 63 °C, the average cooking rate was 3.01 °C/min (range 1.86 °C/min to 4.21 °C/min). For 71 °C, the average cooking rate was 2.61 °C/min (range 1.43 °C/min to 4.13 °C/min). Although the magnitude of differences in cooking rate appears to be relatively small, the extreme differences in elapsed cooking time demonstrates the overall impact of cooking rate on boneless pork chops. To put this range of cooking rates into perspective, a chop with an initial temperature of 3 °C and a cook rate of 2.5 °C/min would reach 63 °C in 24 min, while a chop with the same initial temperature and a cooking rate of 3.5 °C/min would reach 63 °C in 17 min. Therefore, despite using a single cooking method and chops of equal thickness, cooking rate varied between chops (Figure 1). This resulted in significant variation in total cooking time required to reach a targeted temperature.

### 3.1. Correlations among Cooking Rate, Quality Factors and Chop Characteristics

Correlation coefficients were calculated to determine relationships between pork loin or chop traits and cooking rate (Table 2). Cooking rate was not correlated to pH, visual color subjective firmness scores, L*, a*, or b* (r ≤ 0.05, *p* ≥ 0.37) for chops cooked to either 63 °C or 71 °C (Table 2). Cooking rate was weakly correlated with visual marbling scores for chops cooked to 63° (r = 0.09, *p* = 0.04) and 71 °C (r = 0.12, *p* = 0.02). Again, cooking rate was not correlated with initial chop temperature (r ≤ 0.04, *p* ≥ 0.44) for either target temperature. However, cooking rate was weakly correlated with off-grill temperature for chops cooked to 63 °C (r = 0.17, *p* < 0.01) and 71 °C (r = 0.21, *p* < 0.01). Cooking rate was moderately correlated with initial chop weight (r = −0.37, *p* < 0.01) for chops cooked to 63 °C, and weakly correlated with initial chop weight (r = −0.26, *p* < 0.01) for chops cooked to 71 °C.

The correlation between cooking rate and chop weight was not unexpected. Although all chops were cut to 2.54 cm thick, because loin eye area differed between pigs (data not collected) initial weights in chops also differed. Heavier chops would be expected to have come from pigs with larger loin muscle areas and would result in larger surface area exposed to heat during cooking. However, this did not accelerate cooking rate of the chop. In fact, cooking time was related to chop weight in that heavier chops take a longer time to heat [24]. A previous study reported that beef samples that weighed more took longer to cook [25]. The negative correlation between cook rate and initial chop weight supports these prior results.

In general, loin quality traits were not correlated to cooking rate, except that increased marbling was weakly correlated with an increase in cooking rate for both chops cooked to 63 °C and chops cooked to 71 °C. Water has an average thermal conductivity of 0.60 W/m/°C and fat has a thermal conductivity of 0.21 W/m/°C [26]. This suggests that chops with less marbling had more associated water and therefore a greater thermal conductivity score. However, thermal conductivity only explains the ability of heat to pass through a material and not necessarily the rate at which the material heats. To better explain the rate at which a material heats, an understanding of heat capacity is needed. Heat capacity is defined as how much the temperature of a material changes when a certain amount of heat is moved into it [26]. Heat capacity refers to how quickly a material’s temperature changes with the addition of heat. As you add heat to a material with high heat capacity, it will increase temperature more slowly than a material with lower heat capacity. Water has a heat capacity score of 4178 J/kg/°C and fat has a heat capacity score of 2348 J/kg/°C [26]. Because fat has a lesser heat capacity, it will warm more quickly when heat is applied which explains the weak relationship between marbling and cooking rate observed in this population of boneless pork chops. Although it is likely heat capacity rather than thermal conductivity is responsible for the relationship in cooking rate and marbling, thermal dynamic differences of pork chops likely support the hypothesis that compositional differences of fresh pork chops may impact cooking rate.

Marbling and initial chop weight were correlated (r = −0.27 to −0.31, *p* < 0.01, data not provided in tabular form) such that marbling increased as chop weight decreased. Therefore, the correlation between cooking rate and marbling could be a function of differences in chop weight as correlations simply define relationships and should not be used as predictors. Still, [24] concluded, based on heat capacity, and influences of specific heat, that meat with greater fat content would heat faster than leaner meat. Thus, more heavily marbled chops may also heat more quickly regardless of weight, as was indicated in this population of boneless chops.

Despite the lack of correlation with loin quality traits, cooking rate for chops cooked to 63 °C was only weakly correlated with cooking rate for chops cooked to 71 °C (r = 0.33, *p* < 0.0001, data not provided in tabular form). Because temperature fluctuations between and within grills may have influenced cooking time and overall cooking rate, chops were randomly allotted to grills for cooking. Therefore, this poor correlation between cooking rates of chops cooked to different targeted end points was unexpected. However, because these two traits were weakly correlated and the relationship differed from 0 (*p* < 0.0001), it supports the hypothesis that cooking rate differences are, at least partially, inherent to the loin itself and not entirely dependent on outside factors.

### 3.2. Relationships among Cooking Rate, Cooking Loss, and Warner-Bratzler Shear Force

**Figure 2:**
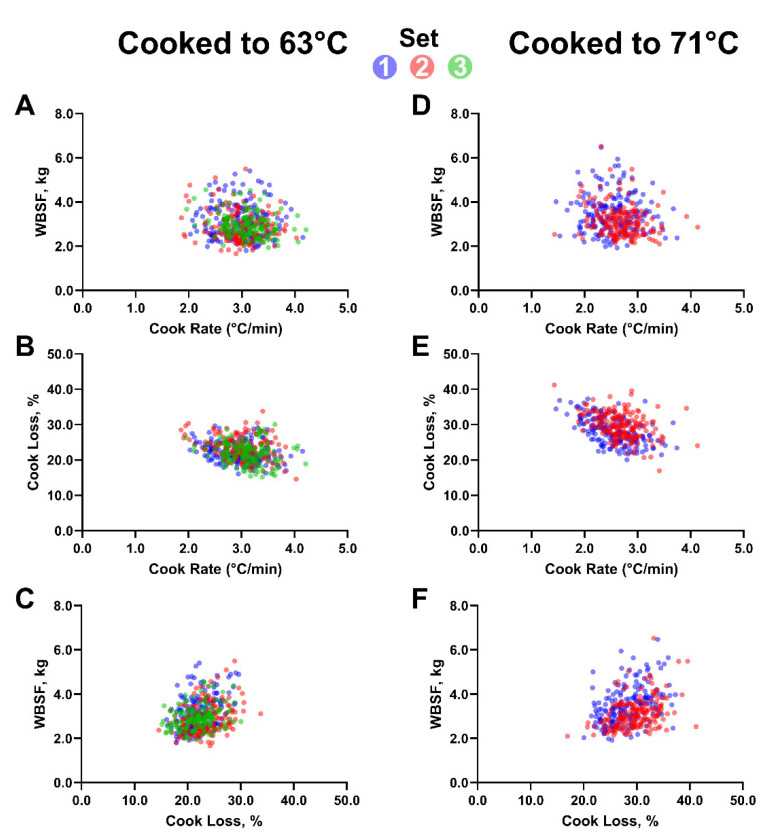
Effect of cooking rate on Warner–Bratzler shear force (WBSF) and cooking loss, and of cooking loss on WBSF. For chops cooked to 63 °C: (**A**) WBSF, kg = −0.201 (cooking rate, °C/min) −0.238 (group 2) −0.146 (group 3) + 3.708; R^2^ = 0.032. (**B**) Cooking loss, % = −0.033 (cooking rate, °C/min) + 0.133 (group 3) + 3.682; R^2^ = 0.076. (**C**) WBSF, kg = 0.076 (cooking loss, %) −0.343 (group 2) –0.169 (group 3) + 1.488; R^2^ = 0.132. For chops cooked to 71 °C: (**D**) WBSF, kg = −0.217 (cooking rate, °C/min) −0.319 (group 2) + 4.025; R^2^ = 0.054. (**E**) Cooking loss, % = −4.512 (cooking rate, °C/min) −0.238 (group 2) + 39.665; R^2^ = 0.256. (**F**) WBSF, kg = 0.085 (cooking loss, %) −0.478 (group 2) + 1.078; R^2^ = 0.201.

In chops cooked to 63 °C, faster cooking rates resulted in decreased WBSF values (*p* < 0.05, β1 = −0.201), such that for every 1°C/min increase in cooking rate, WBSF values decreased by approximately 0.19 kg. However, cooking rate only accounted for 3.2% of the variability in WBSF values (Figure 2A). Similarly, in chops cooked to 71 °C, faster cooking rates decreased WBSF values (*p* < 0.05, β1 = −0.217; Figure 2D), such that for every 1 °C/min increase in cooking rate, WBSF values decreased by approximately 0.22 kg. Cooking rate accounted for 5.4% of the variability in WBSF values of chops cooked to 71 °C. Therefore, increasing cooking rate reduced WBSF and resulted in an improvement in tenderness for chops cooked to 63 °C and 71 °C. However, cook rate only accounted for a small percentage of that variation.

Like the relationship observed between cooking rate and tenderness, an inverse relationship between cooking rate and cooking loss was observed (Figure 2B,E). Faster cooking rates resulted in decreased cooking loss values in chops cooked to 63 °C (*p* < 0.01, β1 = −0.033) and in those cooked to 71 °C (*p* < 0.01, β1 = −4.512). At 63 °C, cooking rate accounted for 7.6% of the variability in cooking loss, but a 1 °C/min increase in cooking rate only reduced cooking loss by 0.33 units. In chops cooked to 71 °C, for every 1 °C/min increase in cooking rate, cooking loss values decreased by approximately 4.51 units and cooking rate explained a greater percentage of variation (25.6%). It is possible that as chops spent more time on the grill and were exposed to heat, muscle proteins had more time to denature and lose water. This would translate into less tender and drier pork chops.

Although there was a statistically significant relationship between cooking rate and WBSF, the overall poor predictive ability may limit applicability, especially within a cooking method. In other words, a substantial change in cook rate would be needed to observe any appreciable effect on tenderness and many other factors also influence cooked tenderness. For example, assuming consumers are generally able to detect a 0.5 kg difference in WBSF values [27], using the relationship observed in chops cooked to 63 °C, an increase in cook rate of 2.37 °C per minute would be needed to observe a 0.5 kg reduction in WBSF values. For perspective, a 2.37 °C per minute change in cook rate would amount to a reduction of average cooking time by approximately eight minutes or about 40%. This dramatic increase in cook rate time for a marginal improvement in WBSF may limit the usefulness of manipulating cook rate for improved pork tenderness.

Contrary to a previous study [7] but in agreement with others [8,9,10], faster cook rates resulted in marginally more tender pork. Faster cook rates also resulted in less cook loss. Potentially, the high heat of the grill used caused a crust to form, thus allowing less water to leave the chop during cooking [28]. Other cooking methods, such as sous vide, with cooler cooking temperatures and no direct heat contact with the meat surface would be less likely to form a crust. In fact, cooking loss from sous vide was greater than using the grilling method of the present study [29]. Therefore, crust formation with different cooking methods that result in differences in cooking losses may warrant further investigation. Because of the previously documented relationships among cooking rate, WBSF, and cook loss [13,28,30], the relationship between cooking loss and WBSF was also assessed. As expected, there was an inverse relationship between cooking loss and WBSF. In chops cooked to 63 °C, greater cooking loss resulted in greater WBSF values (*p* < 0.01, β1 = 0.076), and cooking loss accounted for 13.2% of the variability in WBSF values (Figure 2C). In chops cooked to 71 °C, greater cooking loss resulted in greater WBSF values (*p* < 0.01, β1 = 0.085), and cooking loss accounted for 20.1% of the variability in WBSF values (Figure 2F). It is not surprising that chops that had greater cooking losses had greater WBSF values. Cooking rate did influence cooking loss, although this effect alone does not account for the influence of cooking rate on WBSF. Though there was approximately a 6.5% greater average cooking loss for chops cooked to 71 °C compared with chops cooked to 63 °C, the average WBSF scores only increased by 0.3 kg from 63 °C to 71 °C. This change in shear force between the two DoD would likely not be detectable by consumers as a difference of 0.5 kg is usually required [28].

### 3.3. Prediction of Tenderness Using Quality Traits and Cooking Rate

Coefficients of determination (R^2^) provide information about the “goodness of fit” of a linear regression line and may act as a calculation for the percentage of variation in a dependent variable that can be explained by the independent variable [22]. Using this metric, cook rate accounted for less than 6% of the variability in tenderness in chops cooked to 71 °C and less than 4% of variability in chops cooked to 63 °C. Because cook rate represented a small but significant percentage of variation in WBSF in this study, an assessment of the ability for cook rate to add predictive ability to other factors was performed. Multiple regression with a stepwise selection of independent variables was used to build predictive models for WBSF of chops cooked to 63 °C and 71 °C (Table 3).

For chops cooked to 63 °C, pH was the trait with the greatest partial R^2^ in the final model explaining 19% of variability in WBSF. The β estimate was negative indicating that chops with increased pH resulted in decreased WBSF. In contrast, pH only explained 5% of variability in WBSF for chops cooked to 71 °C though the β estimate was also negative. Historically, chops with a greater ultimate pH had lesser instrumental tenderness values [31,32,33]. However, previous correlations were determined on chops cooked to 71 °C. In a more recent study, pH only explained 5% of the variation in tenderness of chops cooked to 63 °C [34]. The increased β estimate for pH in chops cooked to 71 °C reflects previous research where differences in tenderness between pH categories were more pronounced in chops cooked to 71 °C [35] than those in chops cooked to 63 °C [34]. Cooking loss accounted for 10% of variation in chops cooked to 63 °C and 11% of the variation in chops cooked to 71 °C (Table 3). As expected, the parameter estimate for cooking loss was positive indicating that as chops lost more weight during cooking, a greater WBSF resulted.

Lipid content or marbling was previous correlated with tenderness in pork chops [31,32,33]. Further, it was hypothesized that chops with more lipid and less water would cook more slowly and could differ in WBSF as a result. Composition of pork chops, as measured by lipid content in chops cooked to 63 °C or subjective marbling scores in chops cooked to 71 °C, was influential in predicting WBSF. In each case, the β estimate was negative indicating that chops with more lipid or marbling were less tender, however in either case, these traits only explained 3% or less of the total variability in WBSF. Color of pork chops influenced WBSF. In chops cooked to 63 °C, L* explained 4% of variability in WBSF with lighter chops resulting in reduced WBSF. L* explained 6% of variability in WBSF for chops cooked to 71 °C, again with lighter chops having reduced WBSF. Additionally, subjective color score and b* were also significant factors in the model for WBSF of chops cooked to 71 °C explaining 2% and 7% of variability, respectively. Previously, only 17% of variability in WBSF was explained by L*, a*, and b* in pork chops cooked to 71 through 75 °C [36].

For both temperatures, sire line was a significant factor in the model explaining 1% and 5% of variability in 63 °C chops and 71 °C chops, respectively. Firmness explained approximately 1% of variability in WBSF in chops cooked to either temperature with firmer chops having greater WBSF. While degree of doneness influenced tenderness of pork in both this and previous studies [37,38], cooked temperatures within a degree of doneness only explained variation in WBSF in chops cooked to 71 °C. In total, the temperature when chops were removed from the grill (range 71.0 to 72.5 °C) and the peak final resting temperature (range 67.1 to 74.2 °C) only explained 1% of total WBSF variability. Given the very narrow range of off-grill and final temperatures, their influence on WBSF was surprising.

Overall, the model for chops cooked to 71 °C was able to explain 38% of variability in WBSF compared with the model for chops cooked to 63 °C which explained only 21%. Cooking rate was not a significant factor in either model. Comparing the prediction equations for chops cooked to 63 °C and 71 °C revealed that cooking loss was influential for WBSF at both temperatures, but that pH was more influential than lipid content in chops cooked to 63 °C while lipid content (marbling) exerted more influence than pH for chops cooked to 71 °C. This contrasted with a previous report where marbling was more predictive than pH in predicting eating quality of chops cooked to 63 °C and ultimate pH and marbling equally contributed prediction of eating quality of chops cooked to 71 °C [39]. Therefore, using different combinations of traits may be applicable in predicting tenderness in pork chops cooked to differing endpoint temperatures.

Changes in collagen structure are often cited as reasonings for increased toughness that results as meat is heated from refrigeration to cooked temperatures [40]. While variation in collagen content has been associated with variation in tenderness, these differences are often related to differences in animal age, muscle location, and the sex of the animal [41,42]. Collagen content can vary in pork chops, but this variation has not been associated with variations in tenderness in previous works [43,44]. Therefore, while collagen content was not measured in the present study, it would not be expected to add additional predictive ability to either model.

## 4. Conclusions

Overall, compositional differences of pork chops were related to differences in cooking rate. These inherent differences in cooking rate were independent of outside influencers of cooking rate such as chop thickness, cooking surface temperature, and proximity to heating source. Subtle differences in cooking rate resulted in substantial differences in cooking durations. These data suggest that increasing cooking rate will result in improved tenderness but the predictive ability of cooking rate on WBSF was weak. From a practical standpoint, cooking rate would have to be increased substantially resulting in much shorter cooking times for consumers of pork to detect differences in tenderness. Cooking rate did not add predictive ability to regression models for WBSF. Therefore, while a minor influencer of pork chop tenderness, manipulation of cooking rate is not likely to yield dramatic improvements in pork tenderness.

## Figures and Tables

**Table 1:** Population summary statistics of aged loin quality and cooked chop traits.

Variable		N	Mean	SD	Minimum	Maximum	CV
		Loin Quality					
pH, 14d		503	5.59	0.08	5.39	6.02	1.41
NPPC subjective quality, 14d							
	Color	503	3.47	0.52	2.5	5.0	14.89
	Marbling	503	2.39	0.72	1.0	5.0	30.18
	Firmness	503	2.78	0.40	1.5	3.5	14.23
Moisture, %		405	74.15	0.79	71.34	76.38	1.06
Extractable lipid, %		405	2.37	0.77	0.61	5.44	32.32
Japanese color score		503	2.61	0.66	1.0	5.0	25.02
Instrumental loin color, 14d ^1^							
	Lightness (L*)	503	47.02	2.92	37.05	54.65	6.20
	Redness (a*)	503	6.91	1.12	4.17	11.69	16.20
	Yellowness (b*)	503	5.09	1.16	1.34	8.84	22.81
Purge loss (%), 14 d		503	1.77	1.26	0.08	9.73	71.26
		Cooked Traits, 63 °C					
Initial temperature, 63 °C		503	4.73	1.3	0.3	11.8	27.37
Off-grill temperature, 63 °C		503	63.08	0.27	63.0	67.2	0.43
Final rest temperature, 63 °C		503	66.09	1.08	63.0	70.0	1.63
Cooking time ^2^, 63 °C, min		503	19.75	2.83	13.54	30.32	14.35
Cooking rate, °C/min		503	3.01	0.40	1.86	4.21	13.26
Initial chop weight, 63 °C, g		503	228.96	22.6	168.98	300.71	9.87
Cooked chop weight, 63 °C, g		503	177.57	18.19	129.47	243.6	10.24
Cooking loss (%), 63 °C		503	22.42	3.08	14.58	33.78	13.74
WBSF, 63 °C, kg		503	2.98	0.68	1.66	5.49	22.75
		Cooked Traits, 71 °C					
Initial temperature, 71 °C		357	4.60	1.39	0.8	14.7	30.24
Off-grill temperature, 71 °C		357	71.04	0.14	71.0	72.5	0.20
Final rest temperature, 71 °C		357	72.41	0.74	67.1	74.2	1.02
Cooking time ^2^, 71 °C, min		357	26.09	4.39	16.20	45.47	16.84
Cooking rate, °C/min		357	2.61	0.41	1.43	4.13	15.62
Initial chop weight, 71 °C, g		357	232.89	23.19	175.35	303.04	9.95
Cooked chop weight, 71 °C, g		357	165.43	18.3	117.08	220.78	11.06
Cooking loss (%), 71 °C		357	28.94	3.85	16.94	41.18	13.30
WBSF, 71 °C, kg		357	3.30	0.81	1.92	6.52	24.46

^1^ L* measures darkness to lightness (greater L* value indicates a lighter color). a* measures redness (greater a* value indicates a redder color). b* measures yellowness (greater b* value indicates a more yellow color). Values are from one reading per sample. ^2^ Time elapsed between putting a chop on the grill and taking it off the grill.

**Table 2:** Pearson correlation coefficients (r) between loin quality measurements (14d postmortem) or chop characteristics with cooking rate for chops cooked to 63 °C and 71 °C ^1.^

	Cooking Rate ^5^ for Chops Cooked to 63 °C		Cooking Rate ^5^ for Chops Cooked to 71 °C	
Variable	r	*p*-Value	r	*p*-Value
pH	0.01	0.91	0.09	0.09
NPPC color ^2^	0.01	0.83	0.05	0.37
NPPC marbling ^2^	0.09	0.04	0.12	0.02
NPPC firmness ^2^	0.03	0.53	0.03	0.59
Moisture, %	−0.16	<0.001	−0.02	0.78
Extractable lipid, %	−0.08	0.10	0.08	0.18
Lightness, L* ^3^	0.00	0.99	0.04	0.50
Redness, a* ^3^	0.02	0.64	−0.03	0.51
Yellowness, b* ^3^	0.02	0.62	−0.02	0.66
Initial chop temp., °C ^4^	−0.02	0.60	0.04	0.44
Off-grill temp., °C	0.17	<0.01	0.21	<0.0001
Initial chop weight, g ^4^	−0.37	<0.0001	−0.26	<0.0001

^1^ Loin measures were on the ventral side of the whole boneless loin. ^2^ National Pork Producers Council color, marbling, and firmness scores. Color is measured from light to dark (1–6), marbling is measured least to greatest (1–10), and firmness is measured soft to firm (1–5). ^3^ L* measures darkness to lightness (greater L* value indicates a lighter color). a* measures redness (greater a* value indicates a redder color). b* measures yellowness (greater b* value indicates a more yellow color). ^4^ Initial temperature and weights of chops were taken prior to cooking. ^5^ Cooking rate is measured as the change in degrees Celsius per minute of cooking.

**Table 3:** Stepwise regression predicting traits associated with Warner-Bratzler shear force (WBSF, dependent variable) for chops cooked to 63 °C and 71 °C using fresh and cooked quality loin traits (independent variables).

**63 °C WBSF**	**Mod.**	**Cook Loss**	**Lipid**	**pH**	L*	**Sire Line**	**Firm ^1^**	**Int.**			
R^2^	0.209	0.102	0.034	0.19	0.037	0.011	0.006	--			
β	--	0.056	−0.141	−2.106	−0.05	−0.077	0.138	15.975			
**71 °C WBSF**	**Mod.**	**Cook Loss**	**Marb ^1^**	L*	**pH**	**Sire Line**	b*	**Firm ^1^**	**Color ^1^**	**Temp Final ^2^**	**Int.**
R^2^	0.381	0.108	0.013	0.059	0.045	0.054	0.067	0.012	0.016	0.007	--
β	--	0.06	−0.154	−0.068	−3.999	−0.226	−0.139	0.269	0.249	0.087	20.77

^1^ National Pork Producers Council color, marbling (marb), and firmness (firm) scores. Color is measured from light to dark (1–6). Marbling is measured least to greatest (1–10). Firmness is measured soft to firm (1–3). ^2^ Cooked temperatures measured either when chops were removed from the grill (off-grill) or at the peak of resting temperature (final). Int. is the estimated intercept of the equation. Mod. represents the overall model R^2.^

## Data Availability

Reasonable requests for datasets generated from the current experiment are available from the corresponding author.
